# The Happy Microbe

**Published:** 1903-07

**Authors:** 


### The Happy Microbe.

I'd like to be a microbe,
 And with the microbes play,
Without a task to fret me
 Through all the livelong day.

I'd like to give up striving
 To make ends meet, and fare
Wherever fancy led me,
 Without a worldy care.

I'd like the independence
 To freely come and go,
As does the happy microbe,
 With none to say him no.

The microbe serves no master,
 He never has to sigh
O'er chances that escape him
 Or joys he let go by.

His loving wife ne'er dopes him
  Because his feet get wet;
He needn't go in springtime
  To look for flats "To Let."

At night his mate ne'er greets him
  By running out to say:
"I hope you've had your dinner,
  For Hannah quit today."

His baby never tumbles
  Out of its little bed,
Or crawls beneath the table
  To bump its little head.

House cleaning has no terrors
  That he must ever face;
He never is besmirched by
  A relative's disgrace.

Oh, happy, happy microbe,
  Without a task or care,
I wonder if you envy
  Some smaller mite somewhere?

—*Medical Standard.*

